# Effect of Menadione and Combination of Gemcitabine and Cisplatin on Cancer Stem Cells in Human Non-small Cell Lung Cancer (NSCLC) Cell Line A549

**DOI:** 10.22037/ijpr.2020.112373.13715

**Published:** 2021

**Authors:** Sara Soltanian, Mahboubeh Sheikhbahaei

**Affiliations:** *Department of Biology, Faculty of Science, Shahid Bahonar University of Kerman, Kerman, Iran.*

**Keywords:** Non-small cell lung cancer, Menadione, Gemcitabine, Cisplatin, Cancer stem cells, Cancer stem cell markers

## Abstract

Non-small cell lung cancer (NSCLC) is the most common type of lung cancer. Chemotherapy-induced adverse effects and resistance of NSCLC to conventional drugs reduce the efficacy of current therapies. Tumors contain a small population of cancer stem cells (CSCs) that play a critical role in tumor initiation, maintenance, and drug resistance that finally lead to cancer recurrence. Therefore, CSC-targeting therapies can offer the best hope for developing curative cancer therapies. Vitamins have a high potential for cancer prevention and treatment. Vitamins also ameliorate the side effects which occur in chemo-radio therapy. Menadione (2-methyl-1,4-naphthoquinone/vitamin-K3) is a synthetic form of vitamin K that indicated antitumor activities. The purpose of this study was to evaluate the anti-CSCs effect of menadione and combination of cisplatin and gemcitabine as a first-line treatment in patients with NSCLC on the NSCLC cell line A549. MTT results displayed decreased cell survival after treatment with cisplatin/gemcitabine for 48 h treatment (IC_50_ values 0.25 µM for cisplatin and 5 µM for gemcitabine). Menadione also inhibited the cell growth in A549 cells (IC_50_: 16 µM). Quantitative RT-PCR showed significant downregulation of CSC markers (*Oct4, Nanog, Sox2, Aldh1, Abcb1, CD44, and CD133*) and Snail, epithelial-mesenchymal transition marker, after treatment with menadione and cisplatin/gemcitabine. Flow cytometry showed CD44-positive cells that constitute a high percentage (70%) of A549 cells reduced significantly after treatment with cisplatin/gemcitabine or menadione. However, A549 cells did not show a significant population positive for CD133 and ABCB1 (less than 0.05%), and these fractions did not change after treatment with two agents.

## Introduction

Lung cancer is one of the most commonly occurring cancers and the leading cause of cancer-associated death worldwide (1). Non-small cell lung cancer (NSCLC) is the most common type of lung cancer that is resistant to chemotherapy. Most NSCLC patients respond poorly to conventional chemotherapy (2). Several studies have identified cancer stem cells (CSCs) in NSCLC, responsible for tumor maintenance, therapeutic resistance, and malignant features of this type of lung cancer (3-8). CSCs are a subpopulation of cancer cells within the tumor which are characterized by the ability to self-renew as well as differentiation into multilineage cancer cell types (9). Therefore CSCs express elevated levels of pluripotent stem cell markers, OCT4, NANOG, SOX2 (10, 11). This subpopulation of cancer cells has been found to hold intrinsic resistance to radio-chemotherapy through high aldehyde dehydrogenase (ALDHs) enzyme activity that catalyzes the detoxification of a wide spectrum of drugs (12, 13), enhanced ability for DNA repair (14-17), overexpression of ATP-binding cassette (ABC) transporters which mediate the efflux of multiple chemotherapeutic drugs (18-21) and escaping the effects of the drug via quiescence (22-28). Considering the role of CSCs in cancer therapy failure, therapeutic strategies that target CSCs may bring new hopes to cancer therapy. 

Vitamins are compounds required for normal physiological function and growth of the body. Vitamins are reported to have an apoptotic and inhibitory effect against various cancers. Therefore, the role of vitamins in cancer prevention and treatment has emerged in the past few decades. Furthermore, it is reported that vitamins involve in the amelioration of side effects that occur in chemotherapy and radiation therapy (29-31). As a result, the combination of vitamins with traditional chemotherapeutic drugs can potentiate anticancer efficacy and reduce the side-effects of chemotherapy. Vitamin K is a dietary nutrient that is required for blood clotting. Menadione (2-methyl-1,4-naphthoquinone/vitamin-K3), a synthetic form of vitamin K, is used as a component in multivitamin drugs. It is shown that menadione has antitumor activity against colon, cervix, liver, breast, stomach, nasopharynx, lung, leukemia, and lymphoma cancer cell lines (32-37). Moreover, anti-migratory effects of menadione by modulating the expression of epithelial to mesenchymal transition (EMT) markers was demonstrated in human oral cancer cells (38). 

EMT is a process by which immotile epithelial cells lose cell-cell adhesion and obtain migratory and invasive properties, which have been shown to occur during metastasis in cancer progression (39, 40). Signaling pathways that are critical for CSCs self-renewal and maintenance, such as Wnt, Hedgehog, and Notch, are also activated during EMT. Therefore, according to some evidence, cells undergoing EMT enter the CSC state, acquire drug resistance phenotype as a consequence of elevated expression of anti-apoptotic proteins and increased levels of ABC transporters and express stem cell markers (26, 41-45). Hence, due to the inhibitory effect of menadione on EMT, we hypothesized that the menadione might also target the CSCs population. Therefore, in the present study, we compared the effect of menadione with gemcitabine plus cisplatin as first-line therapy in NSCLC on CSC population and expression of CSC markers in NSCLC cell lines A549

## Experimental

*Materials*

We prepared the RPMI-1640 medium from Gibco, Grand Island, USA, and fetal bovine serum (FBS) and penicillin-streptomycin from Biowest, France. 3-(4,5-dimethylthiazol-2- yl)-2, 5-diphenyltetrazolium bromide (MTT) was from Atocel, Austria. For cell treatment, cisplatin (*Platinol*) and gemcitabine (*Gemzar*) were purchased from Vianex S.A, Greece and Sobhan, Iran, respectively, and menadione (Cat. No. M5625) was from Sigma-Aldrich. FITC anti-human CD44 antibody (Cat. No. 560977; BD Biosciences, San Jose, CA, U.S.A), PE anti- Human CD133/2 (clone: 293C3; Miltenyi Biotec, Bergisch Gladbach, Germany), PE anti-Human CD243 (ABCB1) antibody (Cat. No 919405; Biolegend, San Diego, C.A, U.S.A), Propidium iodide (PI) (Cat. No. P4170; Sigma-Aldrich) and 7-amino-actinomycin D (7-AAD) (Cat. No. 559763; B.D Bioscience) were used for flow cytometry. Total RNA isolation kit was purchased from DENAzist Asia, Mashhad, Iran. RNase-free DNase I (Cat. No. EN0521) and M-MuLV reverse transcriptase (Cat. No. EP0441) was from Thermo Scientific, Wilmington, USA. The real-time PCR was performed using Real QPCR 2x SYBR Green master mix (Cat. No. 5000850-1250; Amplicon, UK). Oligo(dT)18 primer and dNTP were obtained from Yekta Tajhiz Azma, Tehran, Iran. 

*Cell culture and treatment*

Human lung cancer cell line A549 was obtained from the National Cell Bank of Iran (Pasteur Institute of Iran, Tehran). They were cultured in RPMI-1640 medium supplemented with 10% FBS, 100 U/mL penicillin, and 100 mg/mL streptomycin and maintained at 37 °C in humidified air with 5% CO_2_. For treatment experiments, A549 cells were treated for 7 days by combination of cisplatin (0.25 µM)/gemcitabine (5 µM) and 16 µM menadione separately. RNA isolation, quantitative reverse transcription PCR (qRT-PCR) and flow cytometry analysis was carried out on untreated and treated cells to evaluate the gene expression level.

*Measurement of cell viability*

**Figure 1:**
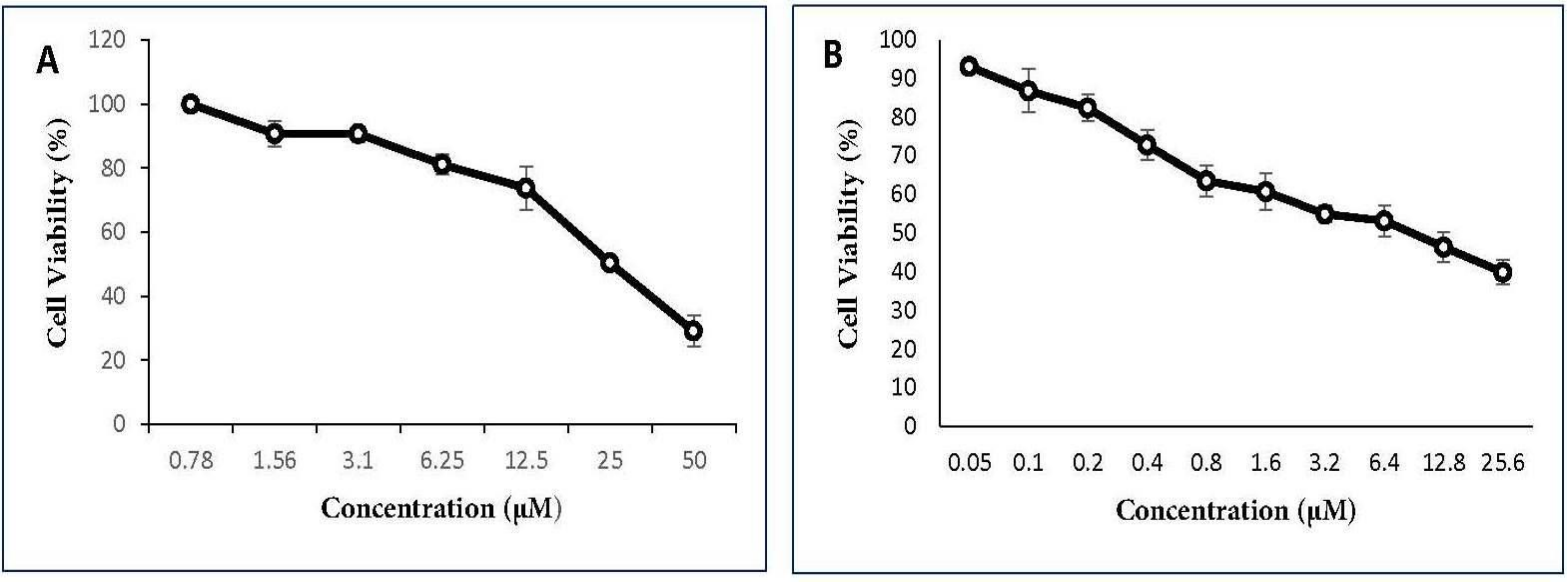
The ani-proliferative effect of cisplatin and gemcitabine on A549 cell line. A549 cell line was cultured and treated with various concentrations of (A) cisplatin (0.78-50 µM) and (B) gemcitabine (0.05-25.6 µM) for 48 h and cell viability percentage was measured. All data are reported as mean ± SD of at least three separate experiments

**Figure 2:**
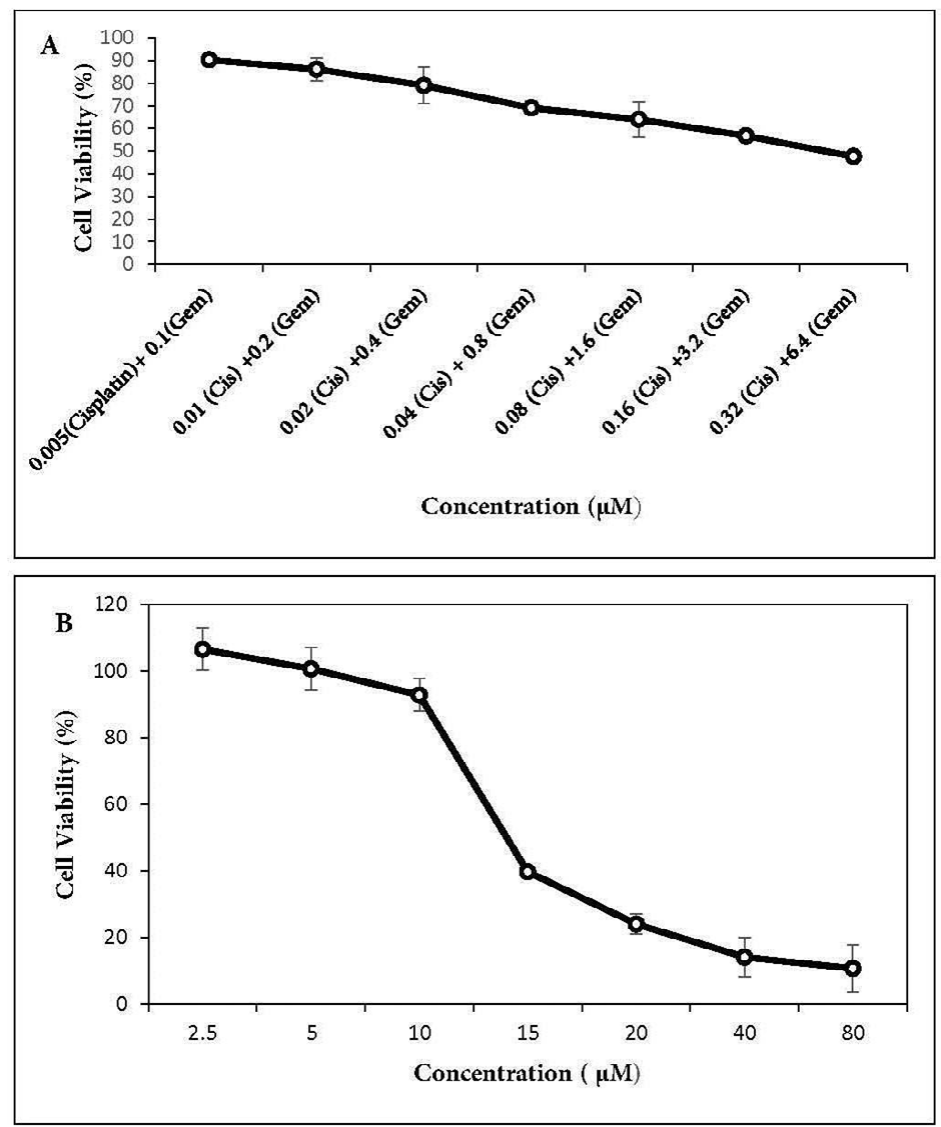
Effect of cisplatin plus gemcitabine and menadione on the growth of A549 lung cancer cells. A549 cells were treated with increasing concentrations of (A) cisplatin plus gemcitabine as well as (B) menadione for 48 h and cell viability was determined by MTT assay. All the points represent results from three independent experiments performed in triplicate. Data are expressed as mean ± SD

MTT (3-[4,5-dimethylthiazol-2-yl]-2,5-diphenyltetrazolium bromide) assay was conducted to evaluate *in-vitro* cytotoxicity of gemcitabine and cisplatin as single agents and their combinations as well as menadione on the A549 cell line. The cells were seeded in 96-plates at a density of 5 × 10^3^ cells/well and allowed to adhere overnight. Cells were exposed to different concentrations of each drug as shown in Figures 1 and 2. After 48 h incubation, 20 µL MTT stock solution (5 mg/mL) was added into each well and cells were incubated for 3 h at 37 °C. Thereafter, 100 μL dimethyl sulfoxide (DMSO) was added to each well to dissolve the formazan crystals. The absorbance was measured at a wavelength of 540 nm by spectrofluorometry (BioTekELx800, USA). The percentage of cell viability calculated as: [(OD 490 treated cells)/OD 490 control cells] × 100. The IC_50_ values of agents were calculated using Prism 6.0 (GraphPad Software, Inc., San Diego, California, USA).

*Quantitative reverse-transcription PCR (qRT-PCR)*

Total RNA was extracted using an RNA isolation kit according to the manufacturer’s instructions and was reverse-transcribed by using M-MuLV reverse transcriptase as described in the protocol. 

Real-time PCR was carried out on Thermocycler (Analytik Jena, Jena, Germany). Each reaction consists of 1X SYBR Green Real-time PCR Master Mix, 1 µL cDNA template, and each primer at 250 nM in a 20 µL reaction volume. Gene-specific primers were designed using Oligo7 Primer Analysis Software. The sequence of primers and product length are described in Table 1. Amplification conditions for *Oct4, Nanog, Aldh1a1, Abcb1, CD133, CD44, Gapdh *and Snail were: 95 ºC for 15 min, followed by 40 cycles of 95 ºC for 20 sec, 64 ºC for 30 sec, and 72 ºC for 15 sec. The same program was used for *Sox2*, except that the annealing temperature was 66 ºC. At the end of the PCR runs to derive melting curves, the temperature was increased in steps of 1 ºC for 5 sec from 60 ºC to 95 ºC. Analysis of melting curves clearly indicated that each of the primer pairs amplified a single expected product with a distinct Tm. The accuracy of the amplification reaction was validated by gel electrophoresis. *Gapdh* was used as an endogenous control to normalize each sample. Relative quantification of mRNA within the samples was examined using the comparative Ct method (ΔCt treated cells – ΔCt control cells = ΔΔC; relative quantity = 2 ^-ΔΔct^). 

*Flow cytometry*

The following protocol was employed to examine the expression of cell surface markers CD44, CD133, and ABCB1 by flow cytometry. Cells were dissociated with trypsin-EDTA and after washing, one million cells were suspended in PBS/2% FBS. The antibodies were added to cells at a 1:5 dilution for FITC-CD44 antibody and 1:10 dilution for PE-CD133 and PE-ABCB1 antibodies for 30 min in the dark. Finally, after washing, cells were suspended in 0.5 mL of PBS/2% FBS and then analyzed by flow cytometry. To detect dead cells, PI was used with FITC-CD44 antibody and 7-AAD was used with PE-CD133 and PE-ABCB1 antibodies. Flow cytometry was done using a FACSCalibur (BD Biosciences, San Jose, CA, USA). Data were analyzed by Flow Jo software, which is provided with the system.

*Statistical analysis*

All data were expressed as Mean±SD and analyzed using SPSS 16.0 software (SPSS Inc, Chicago, IL, USA). Data analysis was performed using Student’s *t*-test, and *P < 0.05* was considered to indicate a statistically significant difference. All the experiments were repeated for 3 times.

## Results

*Dose-dependent antitumor activity of chemotherapy drugs and menadione against NSCLC cell lines A549*

The cytotoxic activities of gemcitabine and cisplatin were first determined individually on A549 cells. As expected, there was a dose-dependent cell-killing effect. IC_50_ values were 22.5 µM for cisplatin (Figure 1A) and 9 µM for gemcitabine (Figure 1B). Co-treatment with two drugs in gemcitabine/cisplatin ratio of 20:1 potentiated antitumor activity. Analysis of cell survival by MTT assay showed that IC_50_ values after 48 h treatment with two drugs were 0.25 µM for cisplatin and 5 µM for gemcitabine (Figure 2A). Menadione also inhibited the growth of the A549 cells with IC_50_: 16 µM (Figure 2B).

*Downregulation of CSC-related genes following cisplatin/gemcitabine and menadione treatment in A549 cells*

**Figure 3:**
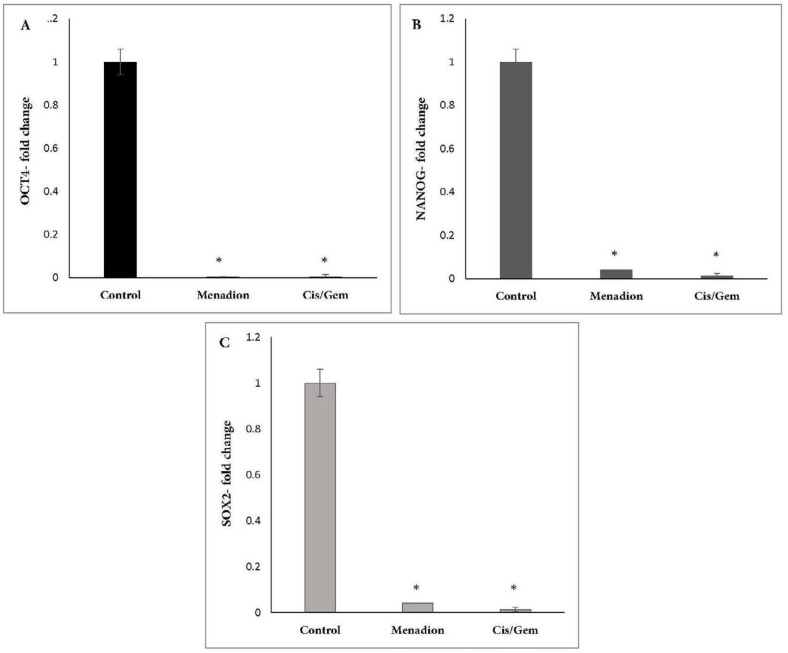
Downregulation of pluripotency transcription factors *Oct4*, *Nanog* and S*ox2* after A549 treatment with cisplatin/gemcitabine and menadione. A549 cells were treated for 7 days by a combination of cisplatin (0.25 µM)/gemcitabine (5 µM) and 16 µM menadione separately and expression of (A) *Oct4*, (B) *Nanog* and (C) *Sox2* were analyzed. Y-axis represents the fold-change in transcript levels compared with untreated A549 cells (designated as 1.0). The graph represents the mean data **± **SD of at least three independent experiments. The asterisk indicates a significant (*p* < 0.05) difference in mRNA expression in comparison with untreated cells

**Figure 4:**
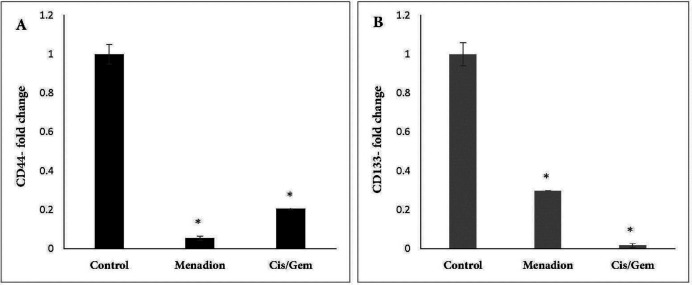
Effect of cisplatin/gemcitabine and menadione treatment on the mRNA expression level of CSC surface markers *CD44* and *CD133* in A549 cell line. mRNA expression levels of (A) *CD44* and (B) *CD133* in untreated control 549 cells were compared to cells treated by cisplatin/gemcitabine (0.25 µM/5 µM) and menadione (16 µM) for 7 days

**Figure 5:**
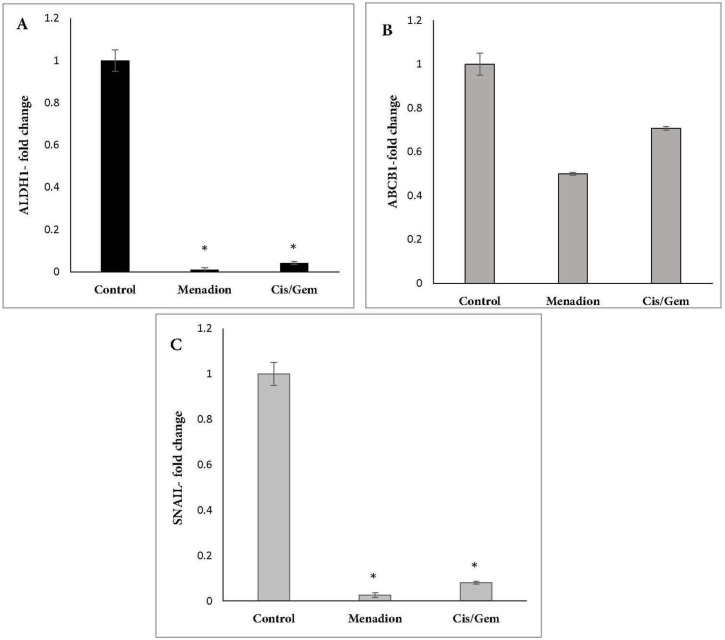
mRNA expression analysis of CSC markers *Aldh1a1*, *Abcb1* and Snail following cisplatin/gemcitabine and menadione treatment of A549. A549 cells were treated with cisplatin/gemcitabine and menadione and transcript expression levels of (A) *Aldh1a1*, (B) *Abcb1* and (C) Snail were measured relative to untreated control cells by real-time PCR

Gene expression analysis of A549 cancer cells treated with cisplatin/gemcitabine and menadione revealed several CSC-related genes and transcription factors that were significantly downregulated after treatment. In detail, stemness-related genes such as *Oct4, Nanog,* and *Sox2 *that control the pluripotency and self-renewal of stem cells showed more than 95% decrease after treatment by cisplatin/gemcitabine or menadione (Figures 3A-3C). Moreover, our data revealed a significant downregulation of *CD44* and *CD133* as well-known CSCs surface markers in response to treatment with both agents (Figure 4). The RNA expression level of *Aldh1a1* as detoxifying enzyme after treatment with cisplatin/gemcitabine and menadione showed 91% and 97% reduction, whereas treatment with both components did not show significant alteration on the expression of drug efflux transporter *Abcb1* (Figures 5A and 5B). Snail is a family of transcription factors that promote the repression of the adhesion molecule E-cadherin to regulate EMT. We demonstrated that the treatment with chemotherapy drugs cisplatin/gemcitabine and menadione decreased the expression of Snails by more than 90% (Figure 5C) (46, 47).

*FACS analysis of CSC surface markers following cisplatin/gemcitabine and menadione treatment in A549 cells*

**Figure 6:**
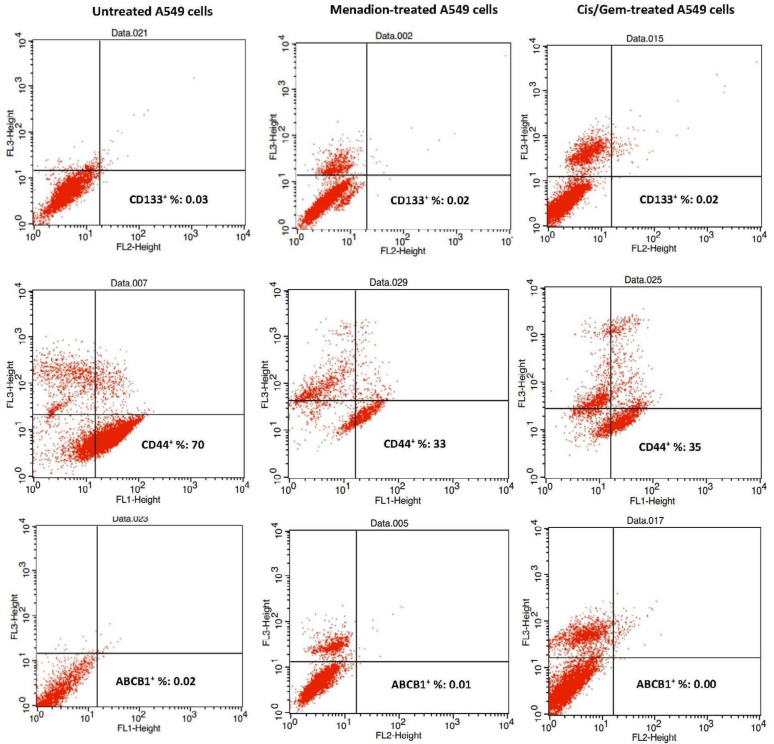
Evaluation of CD133, CD44, and ABCB1 positive cells following A549 treatment with menadione and cisplatin/gemcitabine. A549 cells were treated with a combination of cisplatin (0.25 µM)/gemcitabine (5 µM) as well as 16 µM menadione for 7 days. Cells were analyzed after incubation with PE anti-Human CD133 FITC anti-Human CD44 and PE anti-Human ABCB1 antibodies with flow cytometry. The cell population represented in the right low quadrants are considered positive live cells for each marker

CD133 and CD44 are recognized as robust markers for CSCs in solid tumors (48-50). Therefore, in this study, the expression profile of CD133 and CD44 in cultures A549 cells before and after treatment with cisplatin/gemcitabine and menadione were analyzed by flow cytometry. Data showed that a high percentage of A549 cells expressed CD44 (70%) and this ratio dropped to half after treatment with menadione or cisplatin/gemcitabine. However, A549 cells did not show a significant population positive for CD133 and ABCB1 (less than 0.05%) and these fractions did not alter after treatment with two agents (Figure 6).

## Discussion

Platinum-based doublet chemotherapy is the gold standard therapy for NSCLC patients where cisplatin or carboplatin are used in combination with drugs such as paclitaxel, gemcitabine, or vinorelbine (51). Despite advances in cancer therapy, a low survival rate in NSCLC patients displayed resistance of tumor cells to chemotherapy drugs (52). In various cancer types such as NSCLC, a subpopulation of cancer cells known as CSCs show resistance to therapeutic agents and lead to cancer relapse after therapeutic course (26, 53). On the other hand, traditional anticancer drugs are cytotoxic and inhibit normal cell division. Therefore, fast-dividing body cells such as blood cells and the cells lining the mouth, stomach, and intestines are sensitive to chemotherapy drugs. This results in a range of side-effect and chemotherapy-related toxicities after administration (54). Therefore, low effectiveness of common anticancer drugs against CSCs and their adverse side effects persuaded researcher to develop more effective treatments that could target CSCs and improve the clinical outcome of cancer patients. 

In this study, we showed that menadione (Vitamin K3) reduced the expression of CSCs markers in NSCLC cell line A549. OCT4, NANOG, and SOX2 are three essential transcription factors contributing to pluripotency and self-renewal of normal and CSCs (10, 11). Menadione-induced downregulation of pluripotency markers *Oct4, Nanog*, and *Sox2* show that this vitamin can inhibit self-renewal ability and induce differentiation of CSCs in the A549 cell line. The combination of cisplatin and gemcitabine which is used as a first-line regimen for patients with advanced NSCLC, also downregulated the expression of pluripotent markers. In other studies, downregulation of stem cell-associated genes was indicated after treatment with some chemotherapy drugs such as FOLFOX (5-fluorouracil plus oxaliplatin), docetaxel, and gemcitabine as well as phytochemicals agents such as berberine, apigenin, cinnamic acid, and kaempferol (55-58). 

Moreover, CSCs are identified and isolated from various tumors based on overexpression of specific surface markers (59). CD44 and CD133 are two important surface membrane glycoproteins that have been identified as CSC markers in solid tumors and associate with growth, migration and invasion characteristic, drug resistance, and stem-like properties of CSCs (48, 60-62). In order to assess the effect of cancer cytotoxic agents on CSCs, expression of specific cell surface CSC markers was analyzed in various studies. For example, curcumin and its analog (Diflourinated Curcumin) either alone or in combination with chemotherapy drugs reduced CD44 and CD166 expression in colon and esophageal squamous carcinoma cell lines (63, 64). Here we showed that menadione as well as cisplatin/gemcitabine, meaningfully decreased *CD44* and *CD133* mRNA expression. As a result, it seems that these components can reduce proliferation, tumorigenesis, and stem cell-like properties in A549 cancer cells. Expression of cell surface markers CD44 was detectable on around 70% of the A549 cell line and this ratio decreased significantly after treatment with menadione and cisplatin/gemcitabine that is consistent with qRT-PCR results. However, using our antibody, we were not able to detect CD133 expression on a significant proportion of A549 cells as well as menadione or cisplatin/gemcitabine-treated cells. 

Stem cell populations in many types of tumors are defined by the high activity of ALDHs. ALDHs are a superfamily of enzymes responsible for oxidizing endogenous and exogenous aldehydes to carboxylic acids. Therefore, ALDHs activity confers CSC resistance to antitumor drugs, so components that can reduce the expression of this marker or inhibit its activity play a key role in sensitizing resistant cells to cytotoxic agents (65-69). The current study also confirmed that cisplatin/gemcitabine, a standard drug for lung cancer treatment, and menadione decreased the expression of *Aldh1a1*. Overexpression of the ABC gene family is one of the principal mechanisms for protecting CSCs against many cytotoxic drugs that contribute to cancer resistance to chemotherapy (70). Drug-mediated inhibition in the expression of ABC transporters has the potential to re-sensitize CSCs to chemotherapy agents. Expression of ALDH1 and ABC transports was analyzed in many types of research to determine the effect of anticancer agents on CSCs. For example, expression of ALDH1A1 in the human esophageal squamous cell line was diminished after treatment with curcumin (63). In another research, berberine-treated MCF-7 and baicalein-treated myeloma cells were associated with a decrease in expression of ABCG2 relative to untreated cells (71, 72). Our results did not indicate significant downregulation of *Abcb1* in cisplatin/gemcitabine and menadione-treated cells. Moreover, flow cytometry did not show detectable expression of ABCB1 on A549 cells, and this proportion did not change after treatment with two agents. 

As a key transcriptional repressor of E-cadherin, Snail is recognized as a prominent inducer of EMT and tumor metastatic (73, 74). Considering that migratory cells with EMT phenotype have CSC characteristics, reduced expression of E-cadherin and increased expression of Snail can be considered as markers for detection of CSC population cells. (42, 43, 75). In research on human oral cancer cells, it was demonstrated that menadione reduced metastatic potential by expression induction of E-cadherin and down-regulation of EMT markers such as vimentin and fibronectin (38). Our results also revealed that Snail was downregulated significantly by menadione treatment. Reduced expression of EMT markers shows that menadione can suppress stem-like properties of cancer cells. Consequently, menadione, a synthetic version of vitamin K, exhibited anti-CSCs activities against lung cancer cells. 

The promising role of vitamins, mostly vitamin A and D, in cancer prevention and treatment has emerged from the past few decades (29). Furthermore, vitamin A and D have also been shown to target CSCs in several studies. RA (used simplified here for all-trans-retinoic acid) is a metabolite of vitamin A1 that mediates the functions of vitamin A1 required for growth and development. RA- induced differentiation of CSCs in acute promyelocytic leukemia (APML) and breast cancer show the potential of vitamin A to eliminate CSCs (76, 77). In addition, RA decreased the expression of CSC markers (CD44 and ALDH) and stemness genes (KLF4 and SOX2) and inhibited CSC properties such as tumorspheres formation in gastric cancer (78, 79). β-carotene, a provitamin A carotenoid, inhibited self-renewal characteristics of CSCs and decreased expression of several stem cell markers (80). These findings suggest that RA may be regarded as a chemotherapeutic agent to target the CSC population. Inhibitory effects of vitamin D and its analogs on CSCs in various human cancer cells have been demonstrated in several studies. It was proved that vitamin D could induce differentiation of various neoplastic cells into a more mature phenotype (81-83). Signaling pathways, such as Notch, Hedgehog, Wnt and TGF-β are important signaling pathways in the maintenance of CSCs in human tumors. Therefore, these pathways have been considered as novel targets against CSCs (84, 85). Many studies have reported the inhibitory effect of vitamin D on Notch signaling, Hedgehog signaling and Wnt signaling pathways, indicating vitamin D can be considered as a promising therapeutic/preventive agent against CSCs (83, 86-91). 

In conclusion, tumorigenic ability and resistance of CSCs to conventional chemo- and radio-therapy make them a potential target for anticancer drugs to reduce drug resistance and attenuate the likelihood of relapse. Here we introduced menadione as an anticancer agent that can down-regulate the expression of important CSC markers. Although treatment with cisplatin and gemcitabine showed approximately the same effect as menadione on downregulation of CSC-associated genes, combination therapy with menadione and chemotherapy drugs may not only reduce adverse side effects but improve the effectiveness of chemotherapy drugs and finally achieve prolonged survival of cancer patients. *In-vivo* experiment is needed to achieve more reliable results on the anti-CSCs effect of menadione and further validate the applicability of our results.
